# Supplementary material for: Lactobacillus sp. Facilitate the Repair of DNA Damage Caused by Bile-Induced Reactive Oxygen Species in Experimental Models of Gastroesophageal Reflux Disease
Source: Antioxidants (Basel). 2023 Jun 21;12(7):1314. doi: 10.3390/antiox12071314 (PMC10376144; doi:10.3390/antiox12071314)

Supplemental Figures

Figure S1: Increased Ox Bile exposure reduces esophageal cell viability.
Esophageal epithelial cells were incubated with 0.08 or 0.30% ox bile for 5 minutes, 30 minutes or 48 hours (constant) and 0.08% CA/DCA for 5 seconds, 30 seconds and 5 minutes. Ox bile present for the full 48 hours resulted in reduced cell viability at 24 hours as did incubation with CA/DCA for 5 minutes as measured with CellTiter Blue.

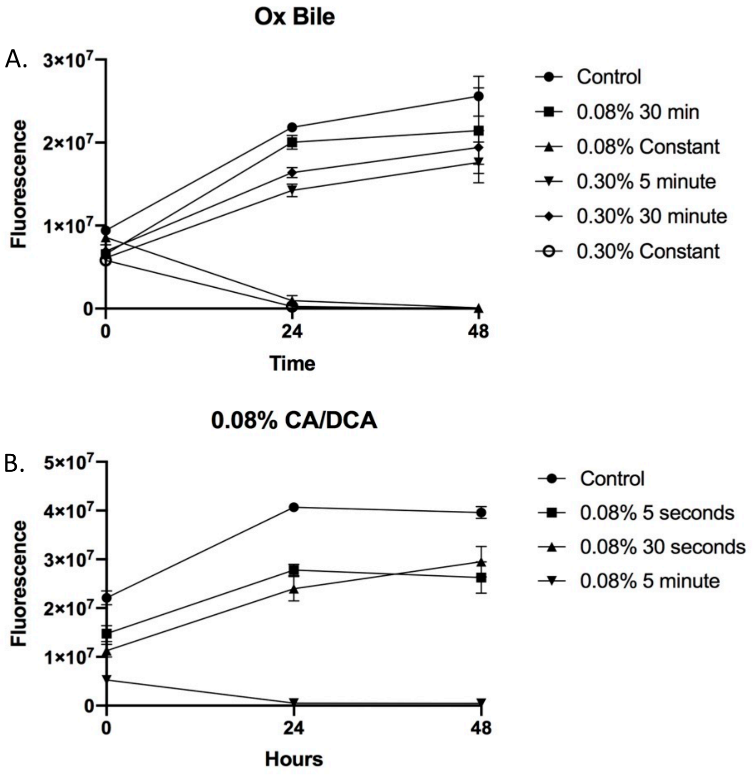

Figure S2: pH2AX is increased after a 30 second ox bile insult and *L. acidophilus* supplemented during recovery. Whole cell lysates were collected before ox bile treatment (0 min) as well as 15 and 30 minutes into recovery in the presence of *L. acidophilus* analyzed by Western Blot using phospho-specific H2AX antibody. Within 15 minutes of recovery, an induction of pH2AX signal in the combination of *L. acidophilus* and ox bile is observed, which is transient as it is diminished after 30 minutes.

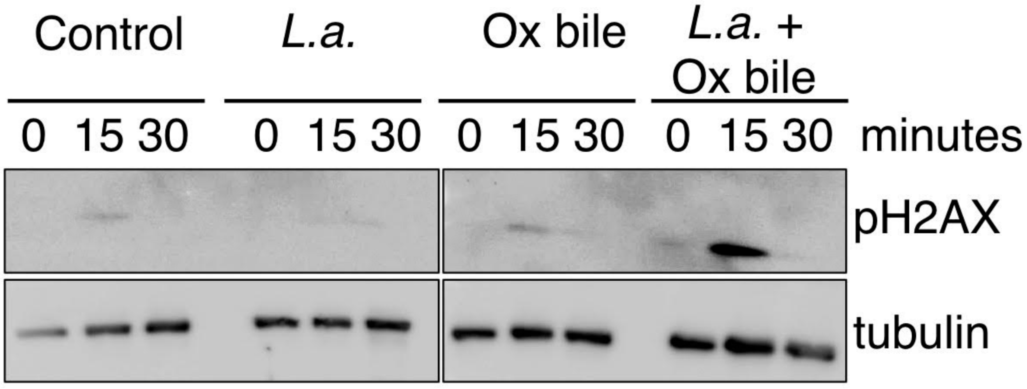

Supplement: Supplementary file 1 [file antioxidants-12-01314-s001.zip › antioxidants-2426051-supplementary.pdf]
